# A longitudinal study of Middle East respiratory syndrome coronavirus (MERS-CoV) in dromedary camels

**DOI:** 10.1186/s12917-023-03769-z

**Published:** 2023-11-02

**Authors:** Mohamed Abdelazim, Rehab Abdelkader, Abdelhakim Ali, Momtaz A. Shahein, Zelalem Tadesse, Ahmed Saad, Amal Mansour, Samah F. Ali, Mohamed Atea, Emma Gardner, Sophie VonDobschuetz, Subhash Morzaria, Yilma Makonnen, Juan Lubroth, Keith Sumption, Ihab ElMasry, Tarek Zakaria, Samah Eid, Eman Abo Hatab, Naglaa M. Hagag, Hend M. Y. Yousef, Mervate Emara, Dina A. Abdelwahed, Hala K. Abdelmegeed, Mervat E. Hamdy, Othman N.O. Mansour, Javier Guitian

**Affiliations:** 1 General Organization for Veterinary Service, Cairo, Egypt; 2 Agriculture Research Center, Animal Health Research Institute, Cairo, Egypt; 3 Food and Agriculture Organizations of the United Nations (FAO), Cairo, Egypt; 4 Food and Agriculture Organizations of the United Nations (FAO), Regional Office for the Middle East and North Africa, Cairo, Egypt; 5 Food and Agriculture Organizations of the United Nations (FAO), Rome, Italy; 6 Institute for Infectious Animal Diseases, Texas A & M University, College Station, USA; 7 Food and Agriculture Organizations of the United Nations (FAO), Sub-regional Office for Eastern Africa, Addis Ababa, Ethiopia; 8 Lubroth One Health Consultancies, Rome, Italy; 9 The Royal Veterinary College, London, UK

**Keywords:** MERS-CoV, Coronavirus, Dromedary camel, Zoonosis, Serology, Longitudinal study

## Abstract

**Background:**

Middle East respiratory syndrome coronavirus (MERS-CoV) was identified in humans in 2012. Since then, 2605 cases and 937 associated deaths have been reported globally. Camels are the natural host for MERS-CoV and camel to human transmission has been documented. The relationship between MERS-CoV shedding and presence of neutralizing antibodies in camels is critical to inform surveillance and control, including future deployment of camel vaccines. However, it remains poorly understood. The longitudinal study conducted in a closed camel herd in Egypt between December 2019 and March 2020 helped to characterize the kinetics of MERS-CoV neutralizing antibodies and its relation with viral shedding.

**Results:**

During the 100-day longitudinal study, 27 out of 54 camels (50%) consistently tested negative for presence of antibodies against MERS-CoV, 19 (35.2%) tested positive and 8 (14.8%) had both, positive and negative test results. Fourteen events that could be interpreted as serological indication of probable infection (two seroconversions and twelve instances of positive camels more than doubling their optical density ratio (OD ratio) in consecutive samples) were identified. Observed times between the identified events provided strong evidence (p = 0.002) against the null hypothesis that they occurred with constant rate during the study, as opposed to clustering at certain points in time. A generalized additive model showed that optical density ratio (OD ratio) is positively associated with being an adult and varies across individual camels and days, peaking at around days 20 and 90 of the study. Despite serological indication of probable virus circulation and intense repeated sampling, none of the tested nasal swab samples were positive for MERS-CoV RNA, suggesting that, if the identified serological responses are the result of virus circulation, the virus may be present in nasal tissue of infected camels during a very narrow time window.

**Conclusions:**

Longitudinal testing of a closed camel herd with past history of MERS-CoV infection is compatible with the virus continuing to circulate in the herd despite lack of contact with other camels. It is likely that episodes of MERS-CoV infection in camels can take place with minimal presence of the virus in their nasal tissues, which has important implications for future surveillance and control of MERS-CoV in camel herds and prevention of its zoonotic transmission.

## Background

Middle East respiratory syndrome (MERS) coronavirus (MERS-CoV) is 1 of 3 major zoonotic coronaviruses to have emerged in the past 2 decades, along with severe acute respiratory syndrome coronavirus (SARS-CoV-1) and severe acute respiratory syndrome coronavirus 2 (SARS-CoV-2) [1]. MERS-CoV was first identified in humans in 2012 in Saudi Arabia [2] and by August 2023, 2583 laboratory-confirmed cases and 889 associated deaths have been reported globally, with a case-fatality ratio of 34.4% [3]. Camels are known to be the natural host for MERS-CoV [4–6] and camel to human transmission of MERS-CoV has been documented [6, 7].

The role of camels as a MERS-CoV reservoir is supported by high prevalence of MERS-CoV antibodies among dromedary camels from African countries (Ethiopia, Kenya, Nigeria, Sudan, Egypt and Tunisia) the Arabian Peninsula (Jordan, Oman, Qatar and United Arab Emirates) and South Asia [4, 8–16]. Cross sectional studies of MERS-CoV in dromedary camels in Africa and Middle East have regularly found high prevalence of MERS-CoV neutralizing antibodies in camel populations. The results of studies attempting virus isolation, usually in nasal swabs, are less consistent, with examples of surveys in endemic populations in which the virus was not isolated despite a large number of camels being tested [17–19]. The relationship between MERS-CoV shedding and presence of neutralizing antibodies in camels is one of several aspects of the epidemiology of MERS-CoV that remains poorly understood despite being critical to inform surveillance and control [20]. Previous studies have demonstrated that camels can shed MERS-CoV even in the presence of neutralizing antibodies [9]. Better insights into the dynamics of viral shedding and antibody levels and their relationship, ideally through longitudinal studies of well-characterized camel populations, can help informing future surveillance and control programs, including future deployment of camel vaccines currently under development [21, 22].

This paper presents the results of a longitudinal study carried out between December 2019 and March 2020 in a closed camel herd in Matrouh Governorate, Egypt, with the aim of characterizing the kinetics of MERS-CoV neutralizing antibodies and the dynamics of viral shedding.

## Results

**Fig. 1:**
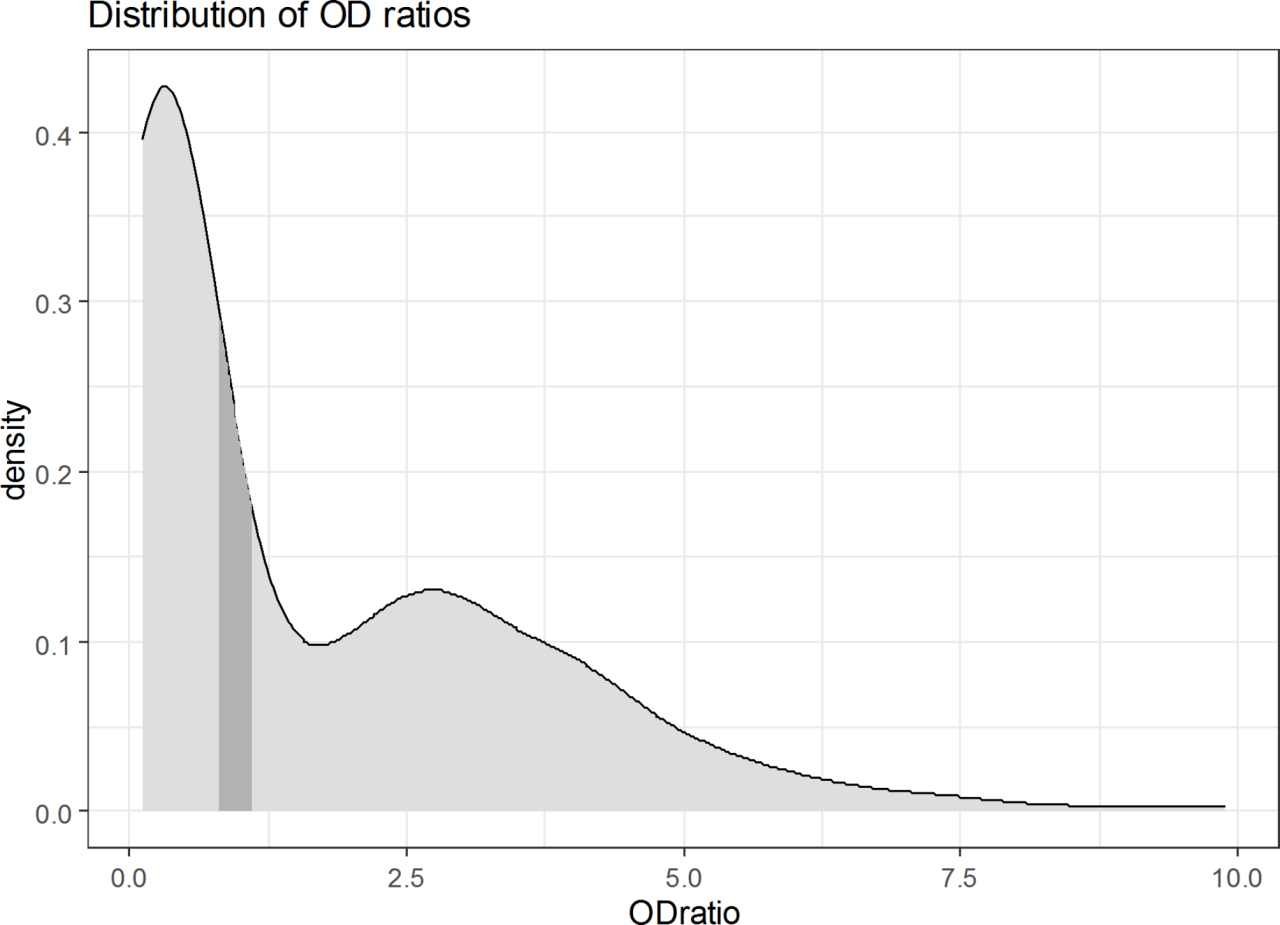
Distribution of optical density ratios (OD ratios) among 540 samples obtained from 54 camels from a single closed herd in Matrouh governorate, Egypt, from December 2019 to March 2020 with sampling carried out 10 times at 10-day intervals. The darker area represents borderline values (≥ 0.8 to < 1.1, as per manufacturer’s instructions)

**Fig. 2:**
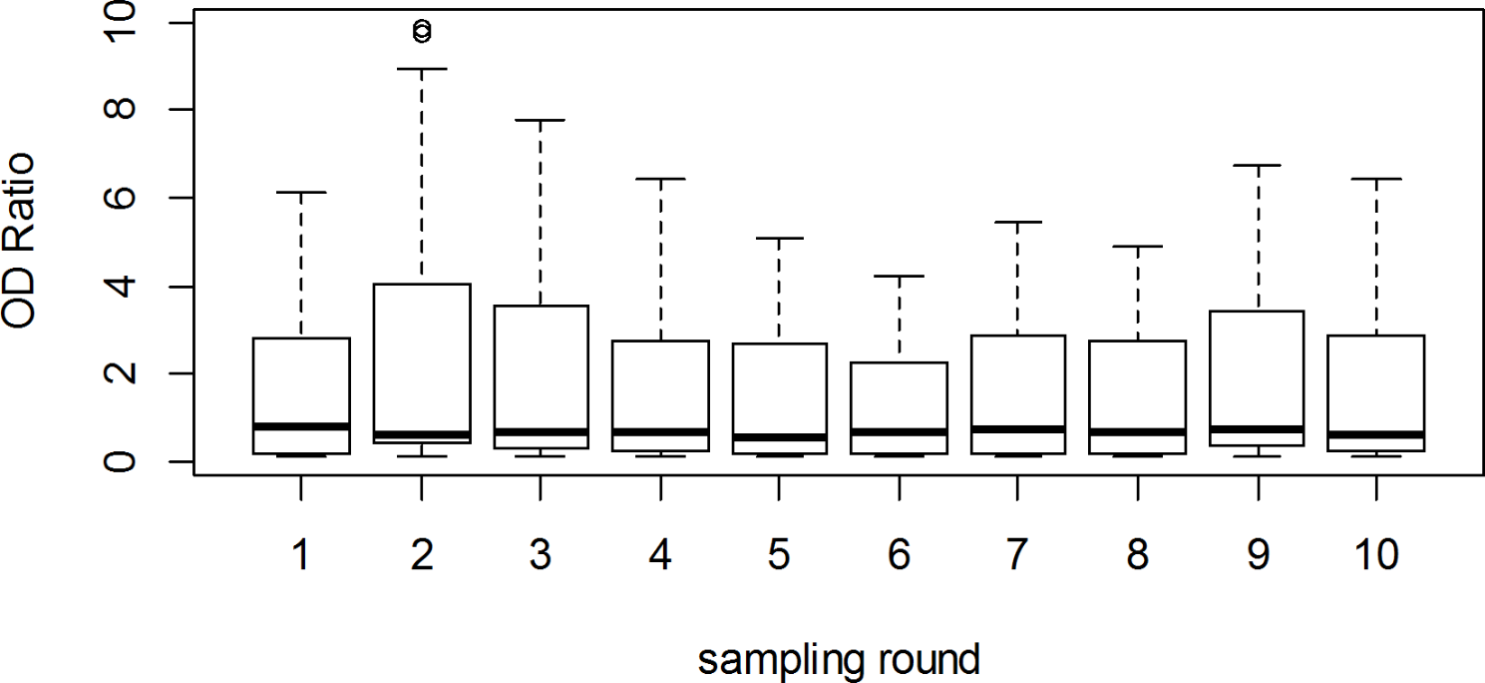
Distribution of OD ratios across 10 sampling rounds from 54 camels from a single closed herd in Matrouh governorate, Egypt, from December 2019 to March 2020. The thick black horizontal lines within the boxes represent the median OD ratio for each sampling round. The lower and upper limits of the boxes represent the 25th and 75th quartiles. The upper “whisker” extends up to the value of the 75th percentile plus 1.5 times the interquartile range. The lower “whisker” extends to the value of the 25th percentile minus 1.5 times the interquartile range

**Fig. 3:**
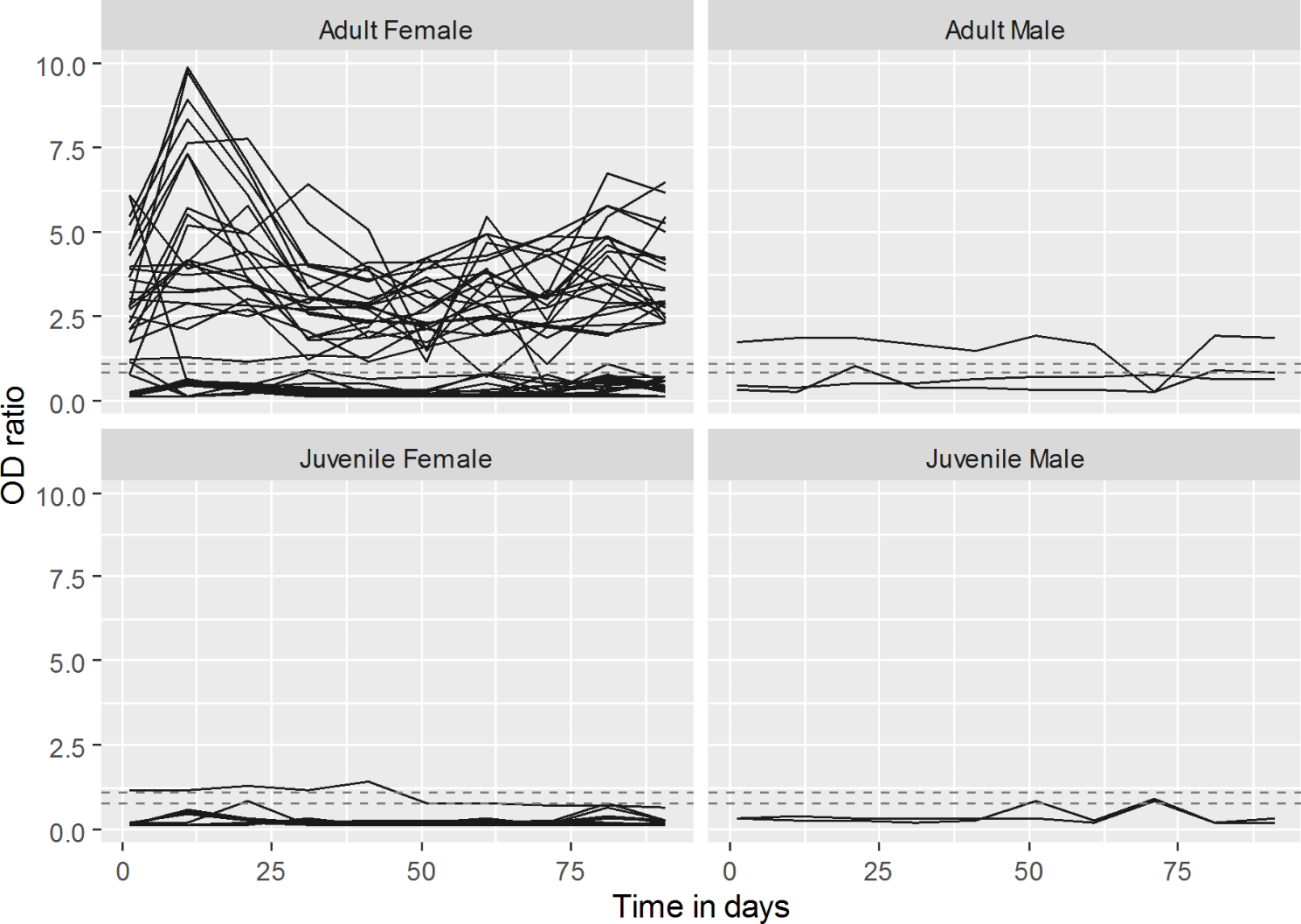
Time series of OD ratios in 54 camels from a single closed herd in in Matrouh governorate, Egypt, from December 2019 to March 2020 with sampling carried out 10 times at 10-day intervals. Results that fall between dashed lines are considered borderline

The distribution of OD ratios across the 540 samples shows a bimodal pattern suggestive of two distinct subgroups and skewed to the right due to a small number of very high values (Fig. 1). The distribution of OD ratios across sampling rounds (Fig. 2) shows that while median values remain relatively stable there is a certain undulation in the highest OD ratios obtained along successive sampling rounds. The time series of OD ratios along the 10 sampling rounds for all individual camels (Fig. 3), shows how adult females, which represent more than 70% of the herd, account for most of the positive test results along the study.

The numbers and proportions of camels found to be positive at the first and last sampling rounds and the numbers showing only positive, only negative or both positive and negative results across all 10 sampling rounds are presented in Table 1, stratified by age and sex. At the time of the first visit, 26 of the camels (48%, n = 54) were found to be seropositive, whereas at the last sampling, 23 of the camels (43%) were seropositive. During the study period, 27 camels (50%) always tested negative, 19 (35.2%) always tested positive and 8 (14.8%) had both, positive and negative test results. Therefore, most of the camels that were seropositive on the first sample tested positive on all samples (19 out of 26). Of the remaining seven camels, four tested positive in nine of the ten sampling rounds, one tested positive in seven, one tested positive in five and two camels tested positive in only one sampling round. Out of the 12 juvenile camels included in the study, only one tested positive (repeatedly in rounds 1 to 5, becoming negative from round 6 onwards).

**Table 1:** Number of camels found to be positive vs. negative on the first sample, the last sample and across all samples by age and sex group (n = 54 camels)

	First sample positive	Last sample positive	All samples		
			**All positive**	**All negative**	**Positive and negative** ^**1**^
Female (49)					
Adult (38)	24 (63.2%)	22 (57.9%)	19 (50%)	13 (34.2%)	6 (15.8%)
Juvenile (11)	1 (9.1%)	0	0	10 (90.9%)	1 (9.1%)
*All female*	*25 (51%)*	*22 (44.9%)*	*19 (38.8%)*	*23 (46.9%)*	*7 (14.3%)*
Male (5)					
Adult (3)	1 (33.3%)	1 (33.3%)	0	2 (66.7%)	1 (33.3%)
Juvenile (2)	0	0	0	2 (100%)	0
*All male*	*1 (20%)*	*1 (20%)*	*0*	*4 (80%)*	*1 (20%)*
*Total*	*26 (48.1%)*	*23 (42.6%)*	*19 (35.2%)*	*27(50%)*	*8(14.8%)*

^1^ Test results classified as borderline were considered negative

The results of the logistic regression for the association of age and sex with odds of being seropositive at the first sampling round showed strong evidence of higher odds of being positive among adult camels, with a sex-adjusted odds ratio (OR) for the association with positive result on first sample of 18.23 (95% CI: 2.14-155.59). The data were insufficient to obtain reliable adjusted estimates for the association with positive result on last sample. Unadjusted associations showed evidence of a higher probability of being seropositive among adult camels (Chi-squared P = 0.0012) and no evidence of association with sex (Fisher’s exact P = 0.38).

**Fig. 4:**
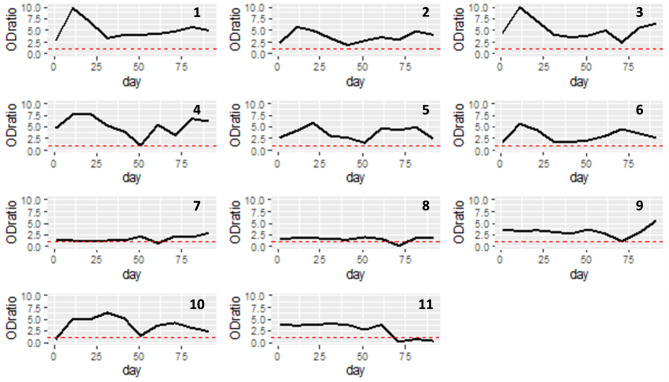
Time series of OD ratios for 11 camels identified as having serological indication of probable infection from a single closed herd in Matrouh governorate, Egypt, from December 2019 to March 2020 with sampling carried out 10 times at 10-day intervals. Camel 10 was negative on the first sampling round, seroconverted in the second and remained seropositive thereafter. Camel 8 seroconverted from negative on sampling round 7 to positive on sampling round 8, after having tested positive in the first six sampling rounds. The remaining camels experienced doubling of their OD ratio in consecutive sampling rounds. The red dashed line represents the threshold for positive test result

We identified 14 events that, according to the assay manufacturer, would be interpreted as serological indication of probable infection. One such event was a camel negative on the first sampling round that seroconverted in the second and remained seropositive thereafter. Another camel seroconverted from negative on sampling round 7 to positive on sampling round 8, after having tested positive in the first six sampling rounds. The remaining events were instances in which positive camels more than doubled their OD ratio in consecutive sampling rounds. Three camels experienced two such events each, therefore the 14 events involved 11 individual camels. The incidence rate was estimated as 2.59 “probable infections” per 1,000 camel-days at risk, considering that camels are at risk of experiencing an infection event even when they are seropositive and allowing for repeated episodes involving the same camel. The time series of OD ratios for these 11 camels is presented in Fig. 4.

**Fig. 5:**
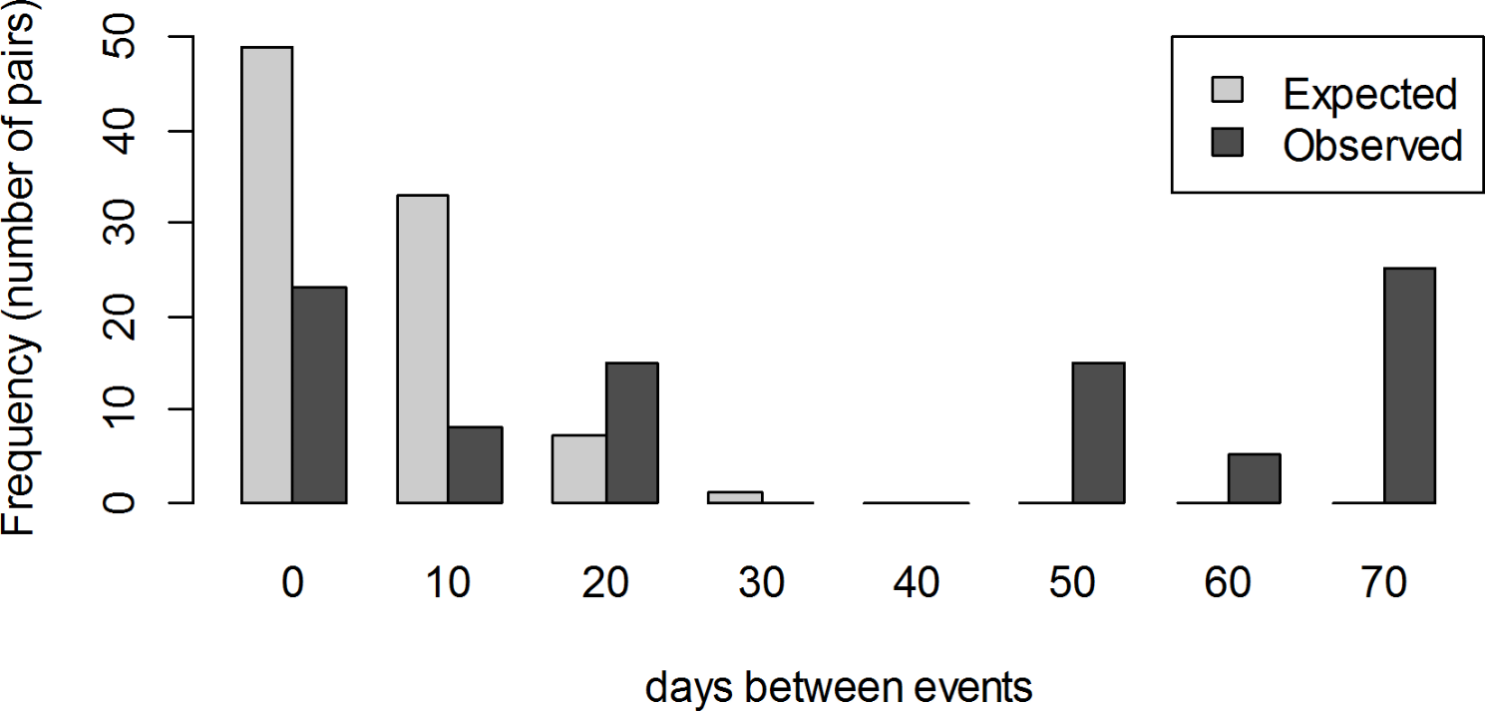
Frequency of observed and expected (assuming X ~ Exponential with rate = 14/9) distribution of times between serological indication of probable infection events (n = 14 events observed in 11 out of 54 camels from a single closed herd in in Matrouh governorate, Egypt, from December 2019 to March 2020 with sampling carried out 10 times at 10-day intervals)

The total number of unique pairs between the n = 14 observed events, calculated as n*(n-1)/2, is 91. The observed inter-event times (observed number of days between the 14 events) are presented in Fig. 5 together with the expected distribution of inter-event times, should the rate of events be constant along the study period. The pattern of observed inter-event times shows a strong departure from the expected distribution if the rate was constant. This is in agreement with the results of the test for equality of mean and variance, which provides strong evidence against the null hypothesis (P = 0.002) that serological indication of probable infection occurred as a homogeneous Poisson process with constant rate during the study period. On the contrary, serological indications of probable infection clustered at specific times.

We did not find evidence of association between age or sex and the odds of serological indication of probable infection (P = 0.56 for age adjusted for sex and P = 0.82 for sex adjusted for age), but the results should be interpreted with caution given the small number of events.

**Fig. 6:**
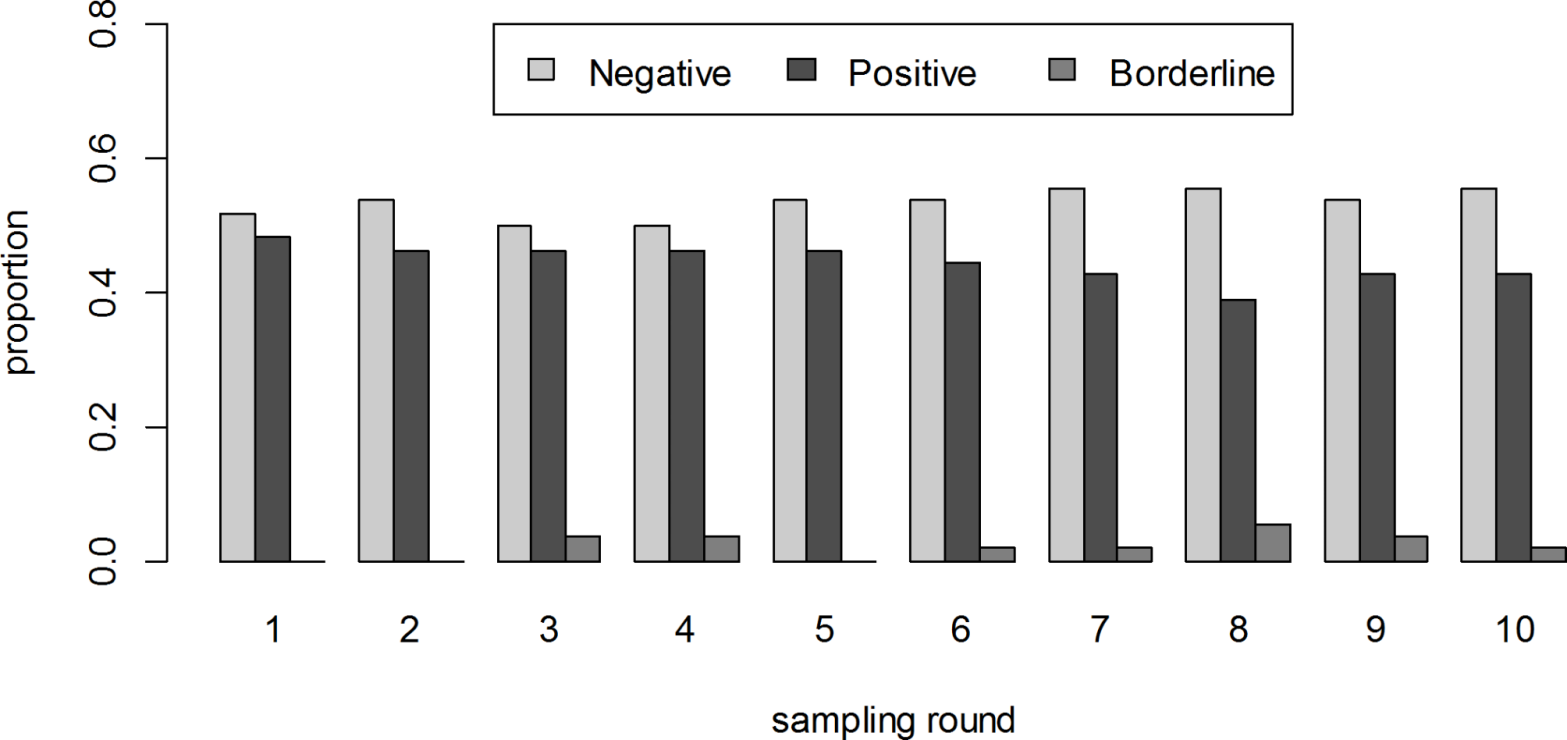
Proportion of negative, positive and borderline serological results by sampling round (results from the longitudinal study of 54 camels from a single closed herd in in Matrouh governorate, Egypt, from December 2019 to March 2020 with sampling carried out 10 times at 10-day intervals)

Despite the large fluctuations observed in some individual animals, the seroprevalence remained relatively stable with a tendency to decline along the whole study from a maximum of 48.1% in the first sample to a minimum of 38.9% in the 8th sample (Fig. 6).

According to the definition of stability that was used (camels that tested positive at least once and in which the OD ratio did not increase by more than 100% or decrease by more than 50% at any point), only 4 of the 27 camels that had positive tests would be considered as having ‘stable’ antibody levels. Thirteen camels experienced both, an increase of more than 100% and a decrease of more than 50% in their OD ratios, ten camels experienced a decrease of more than 50% and no camel experienced only an increase of 100% in their OD ratio without a decrease of more than 50%.

**Fig. 7:**
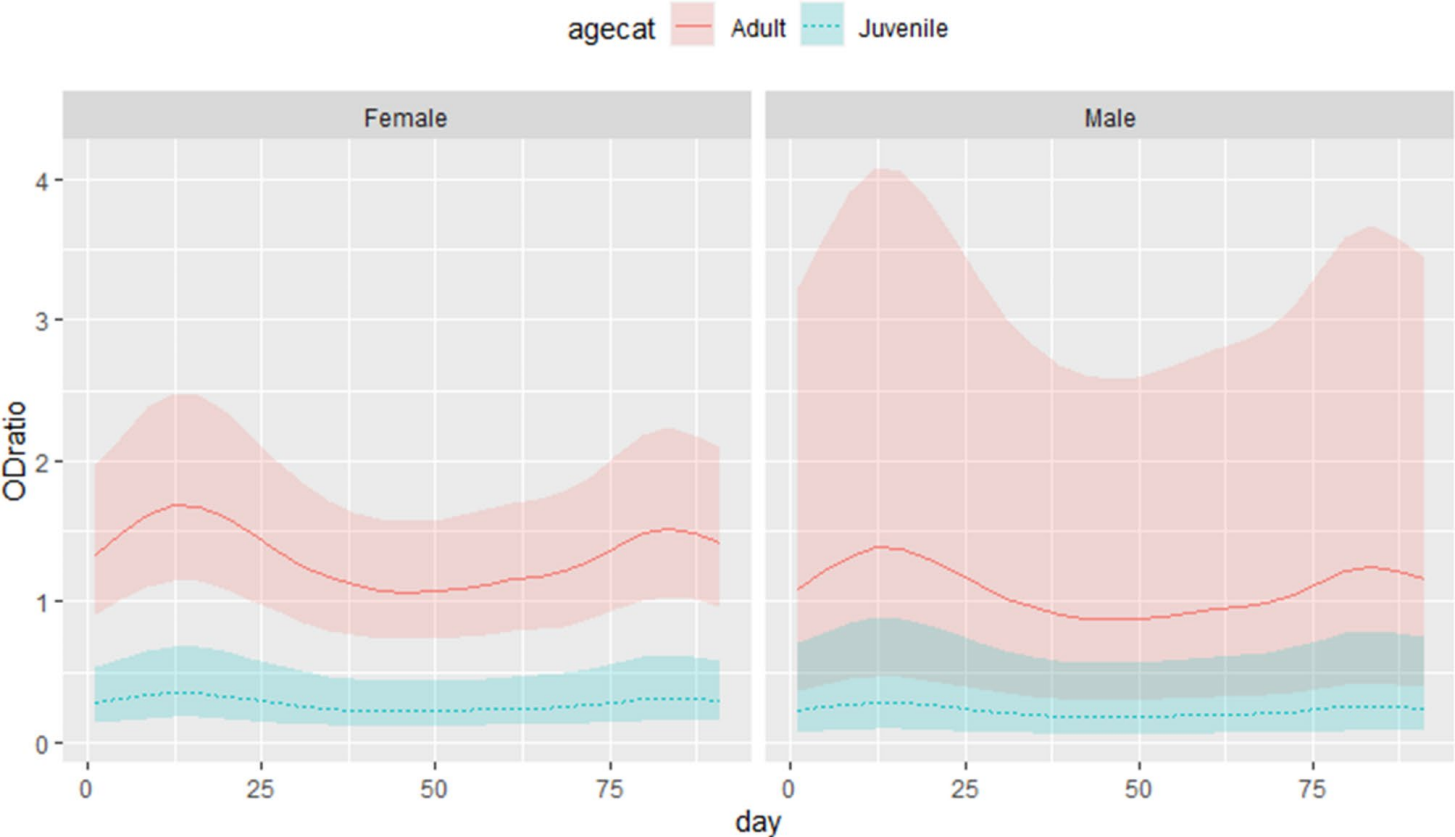
Smooths from the estimates of a Generalized Additive Model (GAM) with effect of time constant across age and sex (results from the longitudinal study of 54 camels from a single closed herd in in Matrouh governorate, Egypt, from December 2019 to March 2020 with sampling carried out 10 times at 10-day intervals)

The results of the generalized additive model are graphically displayed in Table 2. The OD ratio is positively associated with being an adult and there is evidence of variation of OD ratio across individual camels and days, with values peaking around sampling rounds 2 and 9 (Fig. 7).

**Table 2:** Results of a generalized additive model (GAM) of OD ratios with the individual camel as random effect, day as non-parametric smooth term and age and sex as parametric terms

Parametric coefficients		Estimate	SE	P
Intercept		-1.2997	0.3393	0.000145
Age category				
	Adult	1.5773	0.3793	3.8e-05
Sex				
	Female	0.2020	0.5595	0.71829
Non-parametric smooth terms		**edf**	**F**	P*
Day		6.427	6.467	9.76e-08
Individual camel		50.106	42.781	< 2e-16

* Approximate significance

**Fig. 8:**
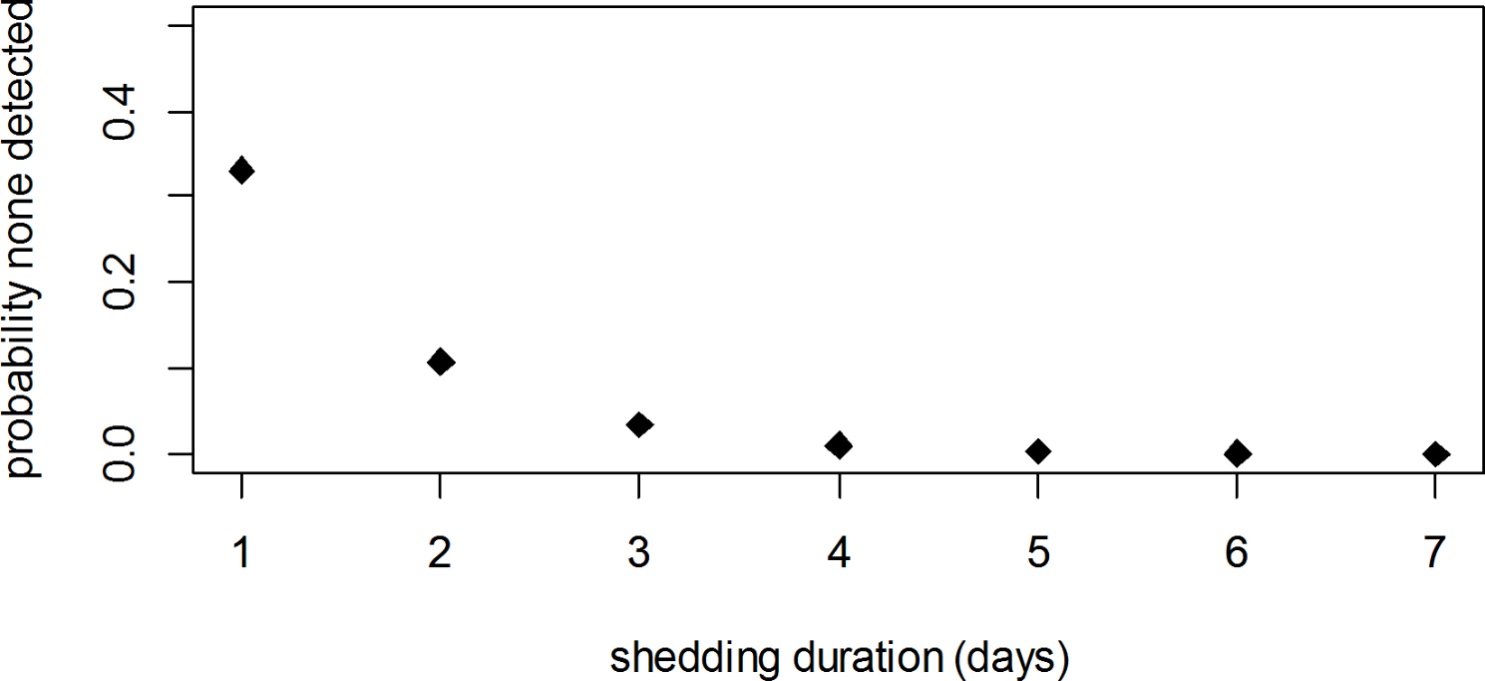
Probability that all nasal swabs from animals experiencing serological indication of infection are negative for different shedding durations

None of the tested nasal swab samples was positive for MERS-CoV RNA, thus analysis of the RNA nasal shedding dynamics was not possible. The probability that all tested samples were negative if there were 11 instances of viral shedding of duration between 1 and 7 days was obtained as the probability of 0 successes out of 110 trials with probability of success of each trial of 1, 2, …, 7 (days) over 100 (length of follow up). The results obtained are only moderately compatible with very short shedding durations of one or two days (Fig. 8).

## Discussion

Longitudinal serological and virological testing of this closed camel herd has provided results that are, to some extent, conflicting. The decline in the proportion of seropositive animals over the last five years is not unexpected. This can be explained by the gradual replacement of old camels by newborn camels and is supported by the lower seroprevalence in juveniles, which can be expected to lose their maternal antibodies 5–6 months after birth [23]. If the serological indicators do in fact reflect infections that occurred during the study period, the negative results obtained for all nasal swabs are highly unlikely unless duration of shedding was very short. Active circulation of the virus within the herd during the study period seems the more plausible explanation of the seroconversion of two negative camels and the marked increase in antibody levels of 9 camels that were already seropositive. However, the study has a number of limitations that warrant caution when interpreting the results. A critical assumption in our study is that a doubling of OD ratios can be interpreted as “probable” seroconversion. Although this interpretation is supported by the manufacturer, we did not test serial dilutions of positive samples, which would have provide additional reassurance of the appropriateness of the interpretation. Furthermore, positive samples were not subject to antibody-virus neutralization test to rule out cross-reactivity. With these limitations in mind, if the results represent a genuine increase in antibody levels in a relatively high proportion of the camels, the more plausible explanation would be re-infection, which has been shown to occur in camels with neutralizing MERS-CoV antibodies [20]. Although this is a closed herd and therefore reintroductions of the virus from camels can be ruled out, the possibility of farm workers bringing the virus into the herd cannot be excluded [24].

The hypothesis that MERS-CoV circulated in the herd during the study is supported by the systematic variation of OD ratios along the study period, with peaks at sampling rounds 2 and 9, as opposed to random fluctuations, and by the clear pattern of inter-event times for seroconversions and marked increases in titers, which clearly departs from what would be expected if these were random fluctuations. It seems reasonable to rule out differences in handling or testing of samples between the different sampling rounds as an explanation for the observed pattern, as the two sampling rounds in which marked increases in individual titers concentrate do not show a general pattern of higher OD ratio across the entire population, with median OD ratios in weeks 2 and 9 lower than for sampling rounds 1 and 7.

Despite indication of probable virus circulation and intense repeated sampling, we were unable to detect MERS-CoV material in any of the 540 nasal swabs collected at the same time as the serum samples. Negative PCR results in high seroprevalence camel populations have been reported elsewhere [17–19] and could be explained by seasonal variations in the intensity of virus circulation and seropositivity being the result of past infections. However, in the presence of active infection in the population, negative PCR results can only be explained by the virus being present in nasal tissue of infected camels during a narrow time window, which has previously been reported [25]. In this study, only extremely short time windows of one or two days would be compatible with the 14 infection events found in 11 of the studied camels. Future studies could include assessment of IgA in the saliva as a means of detection of acute infection.

Other possible explanation for the disagreement between serological and virological findings is the potential latent presence of the virus in an organ rather than the nasal tissues. This hypothesis is tentatively supported by unpublished observations where lymph node tissue from slaughtered camels were MERS-CoV PCR positive, while samples from the nasal cavity were negative (Sophie Von Dobschuetz, 2020, unpublished observations). Persistent infections in a variety of hosts have been documented for other coronaviruses [26]. Finally, cross-reaction with other coronaviruses could potentially explain the misalignment between serological and PCR results. While we think this is unlikely given the high specificity of the serological assay, we cannot rule out cross-reactivity induced by other coronaviruses such as bovine coronavirus as we did not carry out an antibody-virus neutralization test on the positive samples. Assuming that ELISA results were not strongly affected by cross-reaction with other coronaviruses, the study shows highly fluctuating antibody responses against MERS-CoV which are compatible with two periods of increase antibody levels around the second and ninth sampling rounds (days 20 and 90), when most serological indicators of probable infection were detected. No changes regarding distribution and movement of camels within the farm can explain this, which could be due to periods of virus circulation that we were unable to detect by PCR. Alternative explanations could be, as explained above, cross-reactions and inter-assay variation due to changes in handling, processing and testing of samples, but we consider this last explanation to be unlikely.

The study has a number of limitations. First, although this closed herd offered the opportunity of studying in detail a group of camels kept under the same conditions and without contact with other animals, the study population was small and this limited our ability to study differences by gender or physiological status (e.g. gravid or lactating). Furthermore, as indicated above, serological investigations should ideally use additional serological assays and not rely only on one test, in particular, positive samples were not subject to antibody-virus neutralization test and therefore, despite the reported high accuracy of the ELISA test, cross-reactivity induced by other coronaviruses can not be ruled out.

Finally, our assumption that the serological responses identified in this study are in part due to some level of viral circulation during the study period is not supported by the lack of positive PCR results in nasal swabs, occasional brief periods of viral shedding seem to us a reasonable explanation for the serological pattern observed but this hypothesis has to be further investigated.

## Conclusions

Longitudinal serological and virological testing of a closed camel herd with past history of MERS-CoV infection suggests that the virus may have continued to circulate in the herd despite lack of contact with other camels since its last detection five years earlier. The findings are compatible with episodes of MERS-CoV infection in camels taking place with minimal presence of the virus in their nasal tissue, which has important implications for future surveillance and control of MERS-CoV in camel herds and prevention of zoonotic transmission.

## Methods

### Study population and approach

This longitudinal study is carried out in a single closed camel herd at a research farm in Matrouh Governorate (located in the Northwest coastal region of Egypt), from December 2019 to March 2020. The same farm was subject to a previous investigation in 2015–2016 when 93% of camels had MERS-CoV RNA in nasal swabs by RT-PCR [27]. This was followed by a rapid decline in the number of PCR positive camels with no more positive results between July 2015 and February 2016, the time of the last visit.

**Fig. 9:**
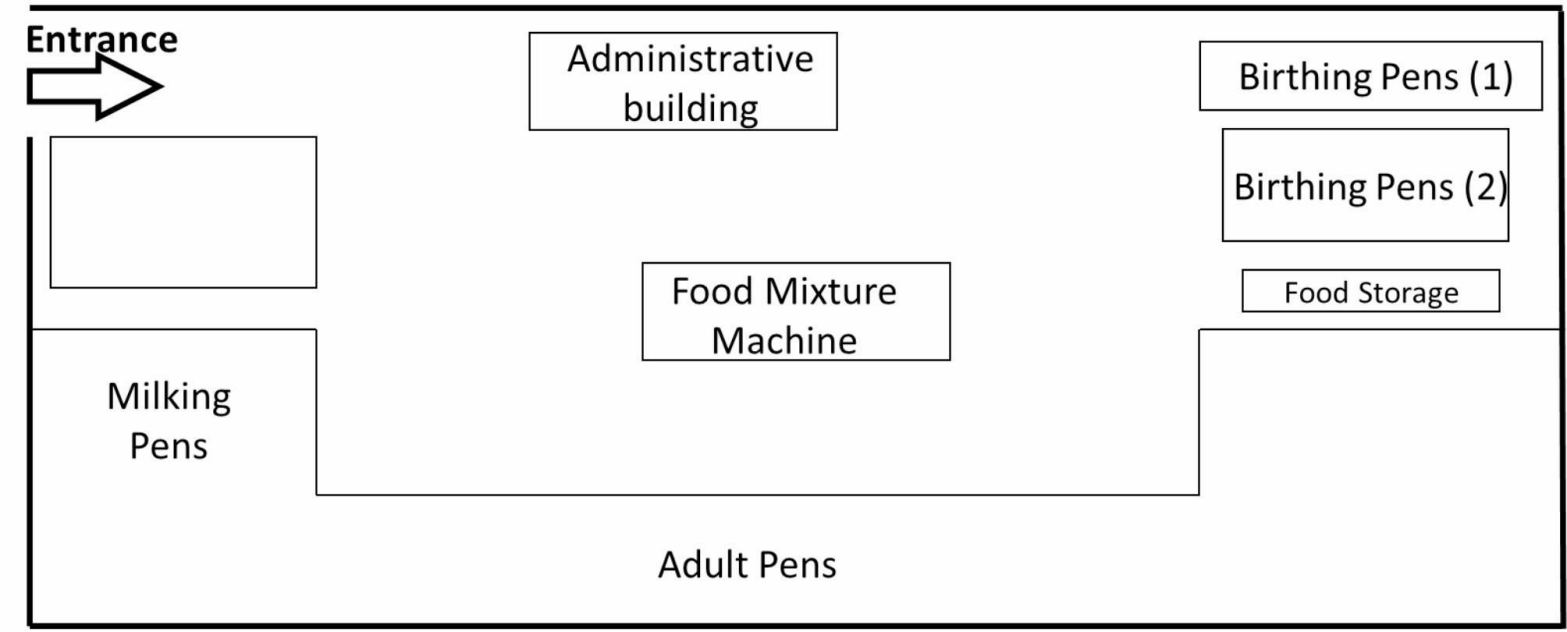
Layout of camel farm in Matrouh governorate, Egypt, where a longitudinal study of MERS-CoV in the camel herd (n = 66 camels) was carried out between December 2019 and March 2020

At the beginning of the current study (26th December 2019), the herd was composed of 66 camels (58 females and 8 males). The last date when camels had been purchased was 2009. In the farm, breeding occurs naturally and there is no contact with animals outside of the farm. The herd can therefore be considered a closed herd. Male camels are occasionally sold to other farms and milk is sold at the farm gate directly to consumers. Camels from this herd did not participate in any events (festivals), the workers of the farm had no regular contact with other camels outside the farm and no animals had respiratory signs on the date of first visit. In the farm, camels use the same feed and water troughs. At the time when the study was conducted, visitors were allowed to enter the camel yard without taking any biosecurity measures. A sketch of the farm layout is presented in Fig. 9 below.

Out of the 66 camels present in the herd at the time of the first visit, 54 were included in the study. Of these, 49 were females (38 adults and 11 juveniles) and five were male (three adults, one juvenile and one neonate). Twelve camels for which it was felt the sampling procedure would be too stressful given their general health and physiological status (pregnancy) were excluded.

During the study period (26th December 2019 to 18th March 2020), sera and nasal swabs were collected from all the 54 camels every 10 days, resulting in a total of 540 samples after the 10 rounds of sampling. Sera and nasal swabs were collected concurrently, on the same day.

### Specimen collection

Blood (10ml) samples were collected by jugular venipuncture in plane vacutainer tubes and sera harvested and coded with the specific animal/sample identification number and date. The sample tubes were kept at + 4 °C and transported to the Animal Health Research Institute (AHRI) using leak-proof transport cool boxes. Nasal swab specimens were collected as per the prescribed procedure [28] and placed directly into pre-labelled 2ml screw top cryovials containing 500 µl of TRIzol®. The cryovials were placed in boxes and kept at + 4 °C for transport.

### Serological testing

All serum samples were tested for the presence of anti-MERS-CoV (S1 subunit) IgG using a commercial IgG rS1-ELISA assay allowing classification of samples as positive, borderline or negative based on their ELISA optical density (OD) ratios. The ELISA assay was carried out and interpreted following the manufacturer’s instructions (rS1-ELISA, Euroimmun) at the AHRI facilities. Accordingly, titer increases exceeding factor 2 and/or seroconversion in follow-up samples were interpreted as possible evidence of acute infection.

### Molecular testing

Real-time reverse-transcription polymerase chain reaction (rRT-PCR) was used to detect the virus genetic material. RT-PCR tests conducted include an assay targeting upstream of the E protein gene (upE) and assays targeting the open reading frame 1b (ORF 1b) and the open reading frame 1a (ORF 1a) [29]. Initial screening was performed using the sensitive upE protocol. Any positive sample was planned to subsequently be tested with the ORF1a, ORF1b or N gene. The cycle threshold cut-off point was determined using a standard curve determination. Molecular testing was carried out at the AHRI laboratory.

### Data analysis

Sequential serological test results were used to derive a number of parameters to monitor the kinetics of anti MERS-CoV antibodies (Table 3).

**Table 3:** Parameters derived from sequential serological testing of 54 camels on 10 rounds at 10-day intervals

Parameter^1^	Level, number of values (unit)	Definition / calculation
Duration of seropositivity	Individual camel level, single value across all sampling rounds (days).	Number of consecutive rounds when camel tested positive*10 days.
Antibody level increase (serological indication of infection)	Individual camel level, 9 values, one for each sampling round except the first (binary event).	When, in the current sampling round, either status of camel changes from seronegative to seropositive or the OD ratio more than doubles compared to that of the previous sampling round.
Number of positive tests	Individual camel, single value across all sampling rounds (discrete number of events from 0 to 10).	Total number of sampling rounds when the camel was found to be seropositive.
Incidence rate^2^	Whole herd, single value across all sampling rounds (cases per camel days at risk).	Number of new “cases” observed during the entire follow-up period over the cumulative sum of camel’s time at risk, with a “case” being an antibody level increase as defined above.
Antibody level stability	Camel, single value across all sampling rounds. Only assessed for camels that tested positive at least once (binary event).	A camel is considered to exhibit antibody level stability if its OD ratio did not increase by more than 100% or decrease by more than 50% during the study period.
Seroprevalence	Sampling round for whole herd (proportion).	Number of camels found to be seropositive at a given sampling round over the total number of camels tested at the same round.

^1^ Borderline considered negative.

^2^ Cumulative sum of camel time at risk obtained assuming that a camel is always at risk of experiencing antibody titer increase.

Chi-square and Fisher’s exact tests were used to assess univariate associations between age group and sex and (i) serological status at the start of the study (sampling round 1), (ii) serological status at the end of the study (sampling round 10) and (iii) serological indication of infection. Adjusted associations of age and sex with each of the three outcomes were assessed by means of logistic regression.

The hypothesis that serological indication of infection events take place randomly along the study period (as opposed to clustered in time) was assessed by fitting a homogeneous Poisson process to the data and testing for equality of mean and variance. The observed inter-event times were obtained and displayed together with the expected inter-event times under homogeneous Poisson process with constant rate along the study period.

A generalized additive model (GAM) with the individual camel as random effect, day as non-parametric smooth term and age and sex as parametric terms was used to model the relationships between these variables and the OD ratio. Restricted Maximum Likelihood (REML) was used to estimate the smoothing parameters.

All analyses were performed in R version 3.6.0, GAM was done using the package mgcv.

## Data Availability

The datasets used and/or analysed during the current study are available from the corresponding author on reasonable request.

## Abbreviations

MERS: Middle East respiratory syndrome
MERS-CoV: Middle East respiratory syndrome coronavirus
OD ratio: Optical density ratio
OR: Odds ratio
SARS-CoV-1: Severe acute respiratory syndrome coronavirus
SARS-CoV-2: Severe acute respiratory syndrome coronavirus 2
